# Interaction between Host and Microbes in the Semen of Patients with Idiopathic Nonobstructive Azoospermia

**DOI:** 10.1128/spectrum.04365-22

**Published:** 2023-01-12

**Authors:** Peigen Chen, Yanqing Li, Xinning Zhu, Menghui Ma, Haicheng Chen, Junxian He, Xiaoyan Liang, Guihua Liu, Xing Yang

**Affiliations:** a Reproductive Medicine Center, The Sixth Affiliated Hospital, Sun Yat-sen University, Guangzhou, China; b GuangDong Engineering Technology Research Center of Fertility Preservation, Guangzhou, China; Tainan Hospital, Department of Health, Executive Yuan

**Keywords:** idiopathic nonobstructive azoospermia, infertility, sperm, seminal microbiota, metabolome, assisted reproductive technology

## Abstract

Men with nonobstructive azoospermia (NOA) face the dual problems of low sperm count and low sperm quality. Most men with NOA without a clear cause are classified as having idiopathic NOA (iNOA). Previous studies found that microbes exist in semen, and the semen microbes of NOA men are different from those of normal men. However, the relevant mechanism is not clear. In this study, we answered the three questions of “who is there,” “what is it doing,” and “who is doing it” by combining 16s rRNA, nontargeted metabolome detection and metabolite traceability analysis. We found that the composition and interaction of seminal plasma microbes in the iNOA group changed. Metabolite traceability analysis and metabolic pathway analysis revealed that microbial abnormalities in the NOA group were closely related to the decrease of microbial degradation of toluene and the increase of metabolism of fructose or mannose. In addition, the metabolic relationship between microbes and the host in male semen in iNOA revealed that such microbes can produce harmful metabolites that affect sperm quality, the microbes compete with sperm for essential nutrients, and their presence reduces sperm production of essential nutrients.

**IMPORTANCE** Idiopathic nonobstructive azoospermia is one of the great challenges in assisted reproductive therapy. Although microdissection testicular sperm extraction technology is currently available, many men with iNOA still face the problem of poor sperm retrieval and poor sperm quality. The role of seminal plasma microbes in male disease has been continuously investigated since semen was demonstrated to harbor commensal microbes. To our knowledge, this is the first detailed description of the microbe-host relationship in iNOA semen. This study is an important complement to research on the treatment and etiology of iNOA and the rationale for our ongoing research.

## INTRODUCTION

Azoospermia, defined as the absence of spermatozoa in the ejaculate in two semen analyses, is observed in 10% to 15% of male infertility patients (1), of which nonobstructive azoospermia (NOA) accounts for about 60% (2). Eighty percent of patients with NOA have no clear etiology and are classified as having idiopathic NOA (iNOA) (3).

The human symbiotic microbiota is closely related to human health. Recent studies have shown that the microbiome can exist in almost every part of the human body (4, 5). Compared with the gut microbiome, the urogenital tract microbiome, although accounting for 9% of the entire human microbiome (6), is still poorly understood. With the development of microbial detection technology, the composition and functional characteristics of microbiota in semen have been gradually recognized (7). The microbiota in semen is associated with male infertility (8). Previous studies have confirmed that microbial changes in semen are closely related to decreased spermatogenesis and decreased sperm quality (9). A study by Massimo Alfano et al. showed that the human testis microenvironment contains small amounts of *Actinomycetes*, *Bacteroides*, *Firmicutes*, and *Proteobacteria* and that the microbiota in semen is associated with complete germ cell hypoplasia in iNOA males. However, the researchers also indicated that the study was preliminary and included a very limited sample size (10).

In this study, we further expanded the sample size and combined it with untargeted metabolome sequencing to fully describe the microbe composition and functional characteristics in iNOA male semen.

## RESULTS

### Subject recruitment.

After rigorous screening, 60 men were recruited in this study, including 30 NOA men and 30 normal men. The basic clinical information of the two groups of men is listed in Table 1.

**TABLE 1:** Basic information for participants^*a*^

Category	Control group	NOA group	*P*
Age (yrs)	32.87 ± 4.932	33.13 ± 5.117	0.838
BMI	23.65 ± 3.207	23.66 ± 2.072	0.9886
Sperm concn (10^6^/mL)	52.21 ± 34.99	0	
PR (%)	49.23 ± 21.31	0	
DFI (%)	18.40 ± 13.30	0	
Basal FSH (U/liter)	3.715 ± 1.264	13.50 ± 8.216	0.085
Basal LH (U/liter)	4.687 ± 1.223	8.390 ± 4.985	0.0903
Basal testosterone (ng/mL)	4.858 ± 1.519	3.753 ± 1.639	0.1542
Basal PRL (ng/mL)	11.49 ± 2.473	15.43 ± 7.875	0.3439
Basal E2 (pg/mL)	24.72 ± 13.43	18.27 ± 10.23	0.3544

a Data are means ± standard deviations. PR, positive rheotaxis; DFI, DNA fragmentation index; FSH, follicle stimulating hormone; LH, luteinizing hormone; PRL, prolactin; E2, estradiol.

### Microbiological characteristics of seminal plasma.

**FIG 1:**
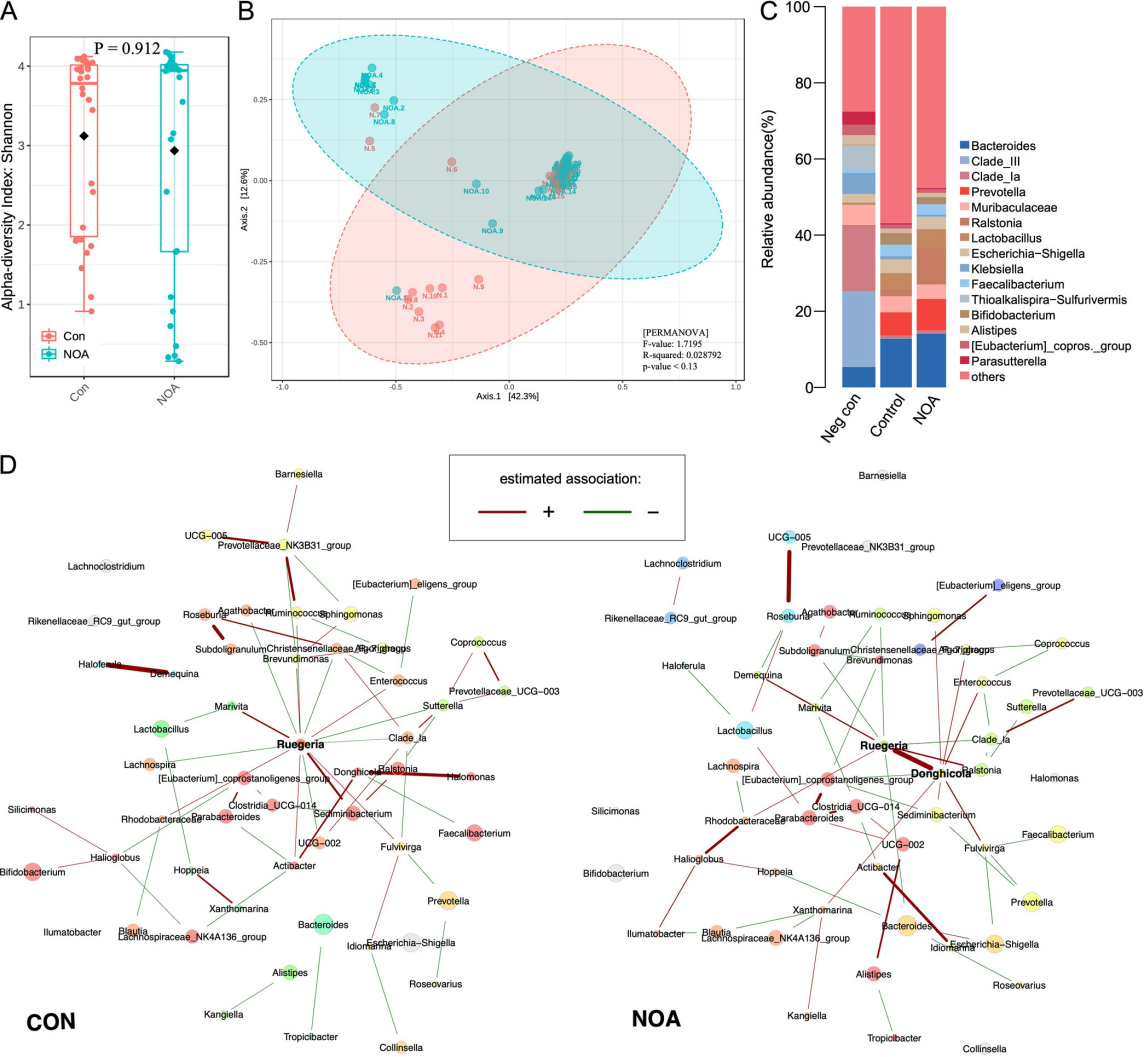
Composition and linkages of the semen microbiota. (A) Boxplots comparing the Shannon index α-diversity of microbial communities in NOA and CON groups. (B) Principal-components analysis plots of β-diversity in the two groups. (C) Stacked plot of genus composition of NOA group, CON group, and negative-control group. (D) The co-occurrence network of the two groups of microbiota suggested that the relationship between the two groups of genera had changed.

After quality control and removal of chimeras, the data volume of valid tags was between 40,762 and 66,832 (see Fig. S1 in the supplemental material). There was no significant difference in α-diversity (Fig. 1A) or β-diversity (Fig. 1B) of microbial composition between the NOA group and control (CON) group. However, the genus composition of the two groups was significantly different, and both were different from the negative-control group (Fig. 1C). Due to the presence of common genera with the negative control in both experimental groups, we subsequently performed decontamination using the decontam R package, resulting in the final removal of 1,000 amplicon sequence variants (ASVs) (Table S1).

### Microbial co-occurrence network and functional characteristics.

**FIG 2:**
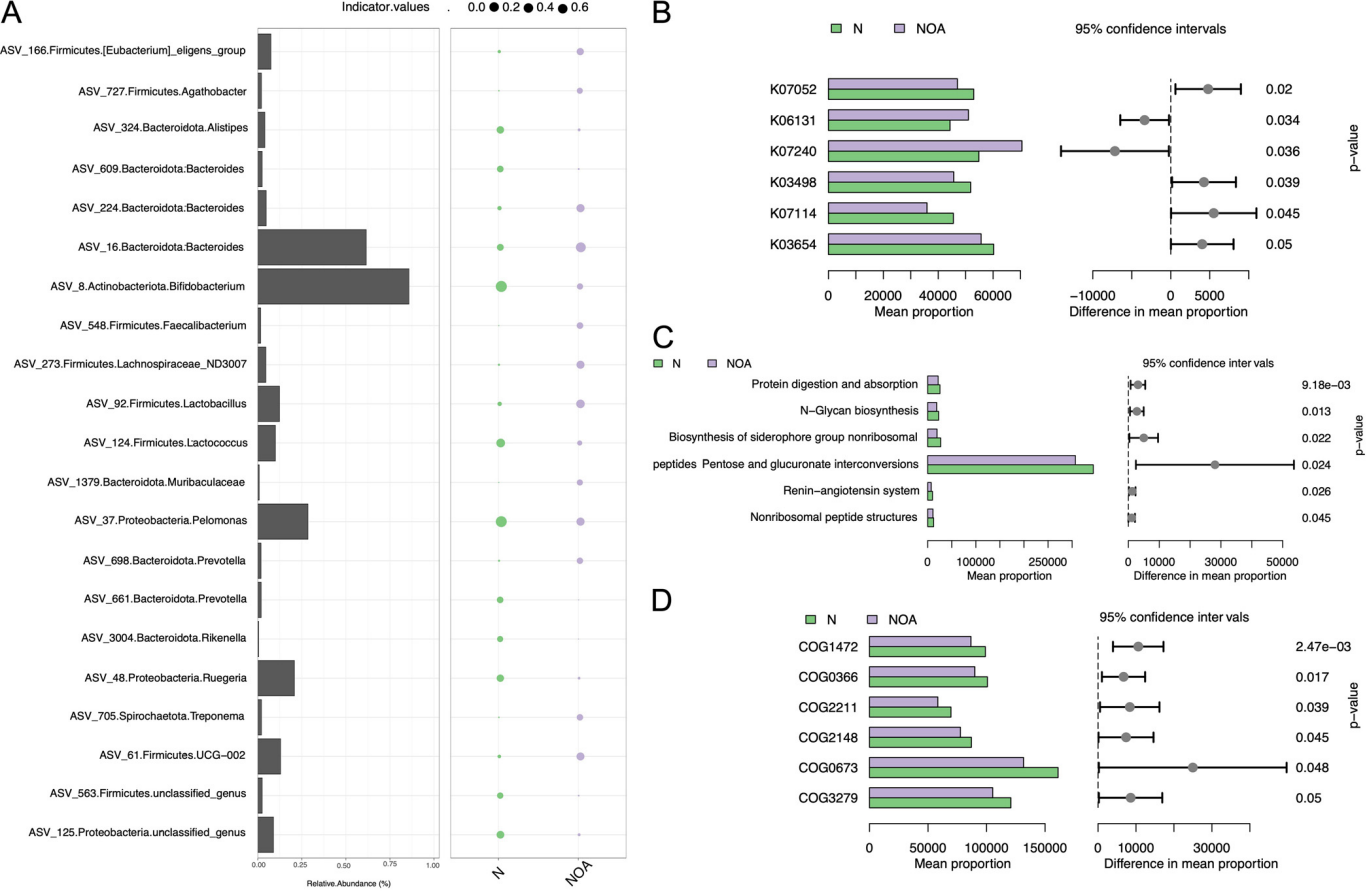
Functional alterations and indicator genera of the microbiota. (A) Indicator genus analysis for both groups. (B to D) Difference analyses of KEGG orthology (B), level 3 KEGG pathway (C), and clusters of orthologous groups (D) between the NOA group and CON group.

Based on the spieceasi method and clr normalization, the co-occurrence network of the top 100 genera with the highest variation in the two groups was constructed (Fig. 1D). The co-occurrence network of the CON group was centered on *Ruegeria*, while *Ruegeria* and *Donghicola* dominated the NOA group. At the same time, the relationships between genera were also significantly different. We simultaneously identified an indicator genus for both groups (Fig. 2A).

In the KEGG Orthology prediction results, the activities of chromate transporter (GenBank accession number K07240) and cardiolipin synthase A/B (EC 2.7.8.-; GenBank accession number K06131) in the NOA group were significantly higher than those in the control group (Fig. 2B). The metabolic pathways of peptides pentose and glucuronate interconversions, biosynthesis of siderophore group nonribosomal, and *N*-glycan biosynthesis in the NOA group were significantly lower than those in the CON group (Fig. 2C). The enrichment results for clusters of orthologous groups of proteins showed the same trend (Fig. 2D).

### Seminal plasma metabolite characterization, source, and functional analysis.

**FIG 3:**
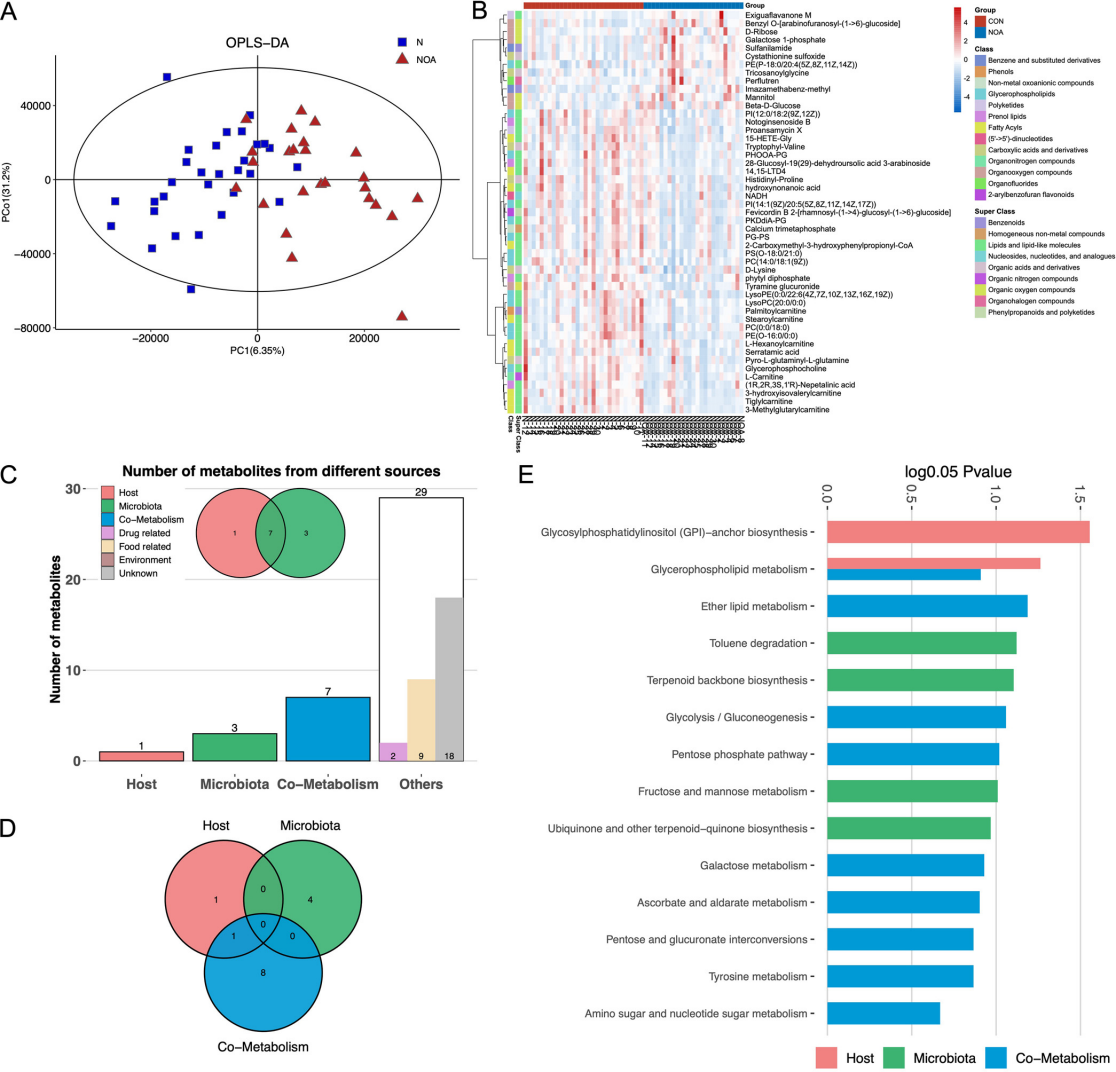
Metabolite composition and traceability analysis. (A) Compositional patterns of metabolites in NOA group and CON group. (B) Heatmap of differential metabolites. (C) Metabolite source statistics. (D) MEPA enrichment statistics. (E) MEPA of metabolites from various sources.

The results of the nontargeted metabolome analysis showed significant differences in seminal plasma metabolites between the NOA group and the CON group (Fig. 3A). Among the differential metabolites, we found that the NOA group had significantly higher expression in the organic oxygen compounds superclass while substantially lower expression of fatty acyls (Fig. 3B).

The functions of metabolites from different sources were analyzed by the metabolic pathway analysis (MEPA) tool (Fig. 3D). Based on metabolite annotation of the HMDB database and MetOrigin metabolite traceability analysis, there were three differential metabolites from microorganisms, seven from microorganism-host cometabolism, and one from the host (Fig. 3C). The metabolites derived from microorganisms were mainly those focused on toluene degradation, terpenoid backbone biosynthesis, fructose and mannose metabolism, and ubiquinone and other terpenoid-quinone biosynthesis. The metabolite functions of microorganism-host cometabolism mainly focused on ether lipid metabolism, glycolysis/gluconeogenesis, and pentose phosphate pathway (Fig. 3E).

### Microbe-host metabolic network.

**FIG 4:**
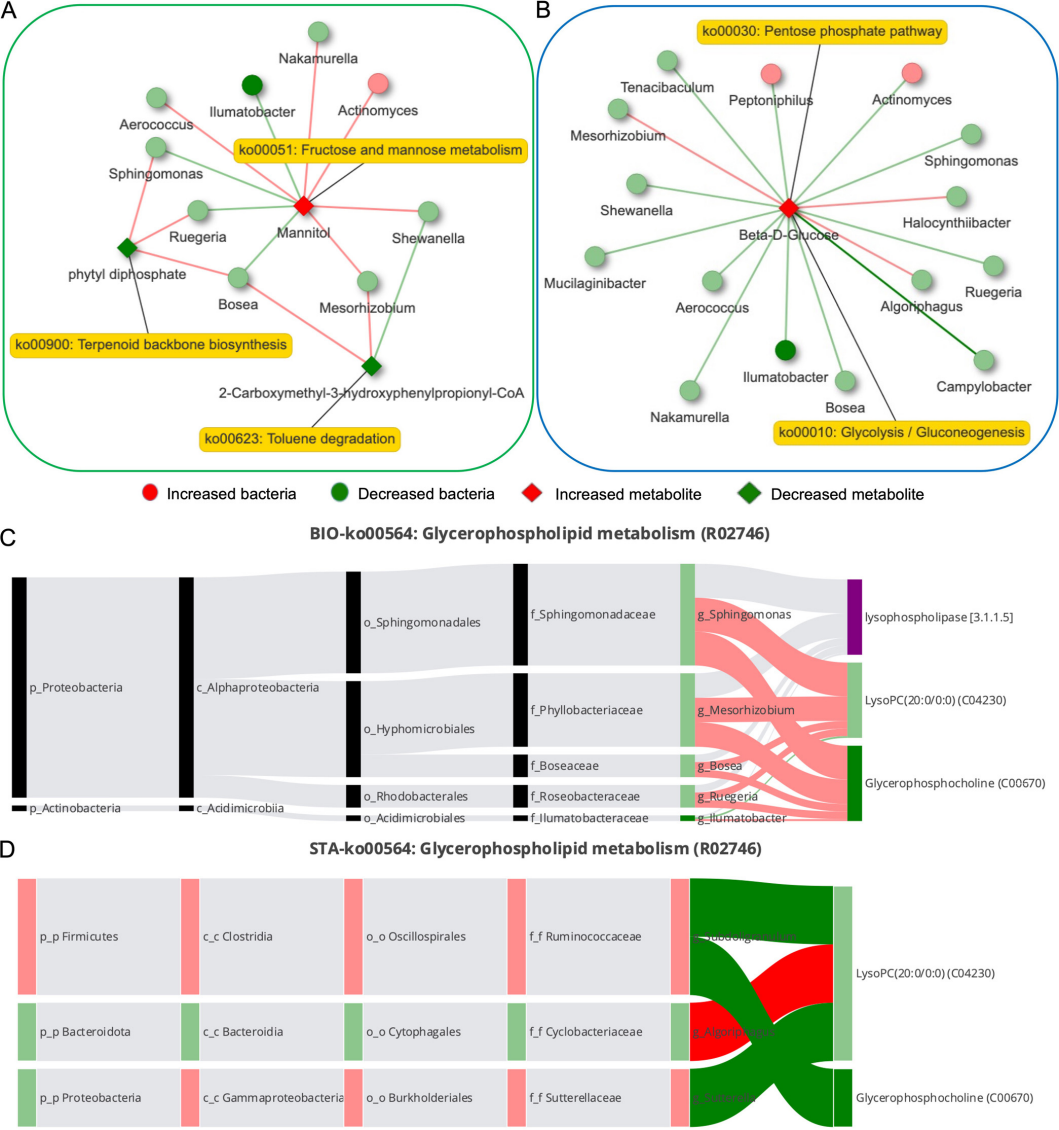
Integrative analysis of metabolites and bacteria. (A) Microbial-derived metabolite network. (B) Host and microbial shared metabolite networks. The diamond and dot shapes represent the relevant metabolites and microorganisms, respectively. (C and D) BIO-Sankey (C) and STA-Sankey (D) network diagrams of the glycerophospholipid metabolism pathway (GenBank accession number R02746).

A microbial-derived metabolite network describes the relationship of mannitol, phytyl diphosphate, and 2-carboxymethyl-3-hydroxyphenylpropionyl-coenzyme A with bacteria. We performed an integrated analysis of metabolites and source genus based on Spearman correlation analysis. Among them, *Bosea* was the only genus that was associated with three metabolites at the same time. *Ruegeria*, at the center of the co-occurrence network, was also associated with mannitol and phytyl diphosphate (Fig. 4A). However, only one metabolite of beta-d-glucose was included in the microbe-host cometabolism network, which various bacteria regulate (Fig. 4B).

At the same time, we explored all potential bacteria that may be involved in the metabolism of the glycerophospholipid metabolism pathway (GenBank accession number R02746) in BIO-Sankey (Fig. 4C) and STA-Sankey (Fig. 4D) networks.

## DISCUSSION

Compared to other fields of microbiome research, the microbial communities found in the male reproductive tract have so far been understudied (7). With the advancement of microbial detection technology, the microbiota in semen has gradually been characterized. It is recognized that it plays a role in male reproductive health and the health of couples and newborns (7, 11, 12).

In this study, we comprehensively characterized the semen microbiota composition, function, and potential effects on sperm of men with iNOA by using 16s rRNA and metabolomic assays combined with metabolite traceability analysis. Since semen is a site of low microbial biomass and is susceptible to microbial contamination from laboratory reagents and the environment (13), we set up three negative controls to identify genuine bacterial sequences.

After removing potentially contaminating sequences, the main genera in semen were *Bacteroides*, *Prevotella*, *Lactobacillus*, *Muribaculaceae*, Escherichia*-Shigella*, and *Faecalibacterium.* The co-occurrence network of the NOA group and the CON group showed that the co-occurrence relationship between the two groups of microbiota changed significantly. The negative correlation between *Ruegeria* and various genera was destroyed, and at the same time, in the NOA group, *Donghicola* rose at the network level, becoming another core genera in the network. In other words, the interaction between members of the NOA microbial community had changed, which might lead to abnormal community metabolic activity and a change in host phenotype. We also found that the indicator genus in the CON group was *Bifidobacterium*, and the indicator genus in the NOA group was *Bacteroides*. *Bifidobacterium* was found to be dominant in high-quality sperm in previous studies (14), and it is also a potential target that can be used to improve sperm quality (15, 16). It has been confirmed in animal experiments that the increase in abundance of *Bacteroides* is significantly negatively correlated with spermatogenesis and sperm motility (17, 18). Meanwhile, in the co-occurrence network, we found that *Bacteroides* and Escherichia*-Shigella*, a potentially harmful genus, were significantly positively correlated in the NOA group.

Metabolite traceability analysis combined with metabolome and microbial information provided a more comprehensive semen microbiome composition and functional landscape. Among the differential metabolites annotated based on the HMDB database, three were derived from microorganisms only, and seven were derived from microorganism-host cometabolism. In the NOA group, microbial degradation of toluene was reduced, and the metabolism of fructose and mannose was increased. Toluene can cause increased DNA fragmentation in sperm, reducing sperm quality (19). Meanwhile, fructose and mannose are important sources of sperm energy metabolism (20). In other words, the abnormal activity of microbes in the semen of the NOA group caused a decrease in sperm quality by increasing harmful metabolites and competing with sperm for energy substances. In addition, the results of metabolic pathway enrichment analysis suggested that glycerophospholipid metabolism was a pathway of microorganism-host cometabolism and was more active in the CON group. Glycerophosphocholine and lysophosphatidylcholine are the main products of glycerophospholipid metabolism and are closely related to the quality of sperm (21–23). In the metabolomic data of this study, glycerophosphocholine was also confirmed to be significantly decreased in the NOA group. In summary, the semen microbiota of iNOA men affects sperm motility and quality through abnormal metabolic activity.

In this study, we answered the three questions of “who is there,” “what is it doing,” and “who is doing it” by combining 16s rRNA, nontargeted metabolome detection, and metabolite traceability analysis. Our results describe the complex metabolic relationship between microbes and the host in iNOA male semen: production of harmful metabolites affects sperm quality, competes with sperm for essential nutrients, and reduces sperm production of essential nutrients. To our knowledge, this is the first detailed description of the microbe-host relationship in iNOA semen. However, the method used to characterize microbial diversity in this study entailed sequencing by PCR-amplifying the V3-V4 region of 16s rRNA. Although the vast majority of bacteria could theoretically be detected, there are still very few genera that cannot be seen in practice, which may lead to underestimating some populations (24). Furthermore, the bias of PCR amplification is well known and inevitable (25). Overall, this study is an important complement to research on the treatment and etiology of iNOA and the rationale for our ongoing research.

## MATERIALS AND METHODS

### Subject recruitment.

All subjects were recruited at the Reproductive Medicine Research Center of the Sixth Affiliated Hospital of Sun Yat-sen University (Guangzhou, China). Each subject voluntarily signed an informed consent form and participated in this study. He or his family members had no genetic disease, systemic disease, long-term exposure to radiation and toxic substances, and no history of drug use. Other inclusion criteria were as follows: (i) no history of STD in the past 2 months; (ii) no systemic corticosteroid use in the past 2 months; (iii) no fever, urinary tract infection, or inflammation in the past 2 months; (iv) no use of over-the-counter or prescription antibiotics, immunosuppressive drugs, systemic corticosteroids, or cancer chemotherapy within 1 month; (v) nO semen infection; (vi) no systemic disease; (vii) not previously diagnosed with cryptorchidism or orchitis.

### Ethics statement.

All study procedures were reviewed and approved by the ethics review board of the Sixth Affiliated Hospital of Sun Yat-Sen University (IRB 2019ZSLYEC-005S).

### Sample collection.

The semen collection method was as described in previous studies (26). Briefly, semen samples were collected by masturbation after 3 to 5 days of abstinence and stored in sterile glass containers. Warm soapy water and 75% alcohol were used for thorough hand and penis disinfection before collection. Samples were stored at −80°C within 2 h of collection.

### DNA extraction and sequencing.

Since semen is an ultralow-biomass site, we set up three negative controls in the study to exclude potential contamination (air, tabletop, reagents) as suggested in previous studies (13).

Microbial DNA was extracted from seminal plasma using the MagPure Soil DNA LQ kit (Magen catalog number D6356-02) following the manufacturer's instructions. DNA purity and concentration were checked by agarose gel electrophoresis. The 16S V3-V4 region (343F, 5′-TACGGRAGGCAGCAG-3′; 798R, 5′-AGGGTATCTAATCCT-3′) was amplified using TaKaRa *Ex Taq* (TaKaRa catalog number RR001Q). The amplified product was purified using Agencourt AMPure XP beads (Beckman Coulter Co., USA) and quantified using a Qubit dsDNA assay kit. 16s rRNA sequencing was performed on the NovaSeq6000 platform in paired-end 250-bp mode (Illumina Inc., San Diego, CA).

### 16s rRNA data analysis.

After trimming the original FASTQ data with cutadapt (27), we used the DADA2 tool (28) to perform quality control analysis, such as quality filtering, noise reduction, splicing, and dechimera according to the default parameters of QIIME 2 (2020.11), and we obtain representative sequences and ASV abundance tables (29). We then aligned the representative sequences with the Silva (version 138) database (30) to annotate genera using the q2-feature-classifier (default parameter).

Contaminants in 16s rRNA sequences were removed using the decontam R package (31) with default parameters. The filtered ASV matrix was used for subsequent analysis. The α-diversity between the two groups was described using the Shannon index, and the Wilcoxon rank-sum test was used to compare groups. β-Diversity was represented using Bray Curtis distances and compared using permutational multivariate analysis of variance. NetCoMi (the Network Construction and Comparison for Microbiome Data) R package (32) was used to construct the co-occurrence network of the two groups. Differential genera between groups were screened using the DEseq2 R package. Indicators from both groups were selected by using the labdsv R package and screened for indicator genera between the two groups with a threshold *P* value of <0.05.

The function of microbiota was predicted using PICRUSt2 (Phylogenetic Investigation of Communities by Reconstruction of Unobserved States) software (33). STAMP software screened functional differences between NOA and CON groups (34).

### Nontargeted metabolome assays.

Metabolite detection was performed using liquid chromatography (LC) and mass spectrometry (MS) (Acquity UPLC I Class plus, Waters). The extraction and detection procedures of metabolites were described in previous studies (35). Briefly, after samples were thawed at room temperature, 1 mg was added to a 1.5-mL Eppendorf tube, and 20 μL of an ice-cold mixture of methanol and acetonitrile (2/1 [vol/vol]) was added. l-2-Chlorophenylalanine was added to methanol as an internal standard. After repeated centrifugation, drying, and filtration, metabolites were extracted. Subsequently, we prepared quality control samples by mixing aliquots of all samples as pooled samples.

After baseline filtering, peak identification, integration, retention time correction, peak alignment, and normalization of raw LC-MS data using Progenesis QI V2.3 (Nonlinear, Dynamics, Newcastle, United Kingdom) software, we determined accurate mass-to-charge ratios (*m/z*) for secondary fragmentation and isotopic distribution for compound identification. For qualitative analysis of metabolites, we used databases such as Human Metabolome Database (HMDB), Lipidmaps (V2.3), Metlin, EMDB, and PMDB.

Peaks with more than 50% missing values (ionic strength = 0) in the resulting metabolite matrix were removed and replaced with half of the minimum value. Compounds with scores below 36 (of 60) were also considered inaccurate and removed. Based on the scores, we merged the positive and negative example matrices.

Differential metabolites between the NOA and the CON groups were analyzed using orthogonal partial least squares discriminant analysis. The quality of the model was assessed using 7-fold cross-validation and a 200-response permutation test.

The overall contribution of each variable to group discrimination was used for ranking based on variable projected importance (VIP) values. A two-tailed Student's *t* test was then used to verify whether the metabolites differed significantly between groups. Metabolites with VIP values of >1.0 and *P* values 0f <0.05 were considered to significantly differ between the two groups.

### Metabolite traceability and integrated analysis.

MEPA tools achieved the functional analysis of metabolites. We then used MetOrigin software to perform metabolite functional enrichment analysis and metabolite traceability analysis (36). MEPA is widely used in metabolomics to identify the most relevant pathways by combining enrichment analysis with pathway topological characterization (37).

### Data availability.

The 16s rRNA gene sequencing results have been deposited with the China National Center for Bioinformation (https://ngdc.cncb.ac.cn/) under reference number PRJCA012767.
