# Supplementary figures and images for: Interaction between Host and Microbes in the Semen of Patients with Idiopathic Nonobstructive Azoospermia
Source: Microbiol Spectr. 2023 Jan 12;11(1):e04365-22. doi: 10.1128/spectrum.04365-22 (PMC9927269; doi:10.1128/spectrum.04365-22)

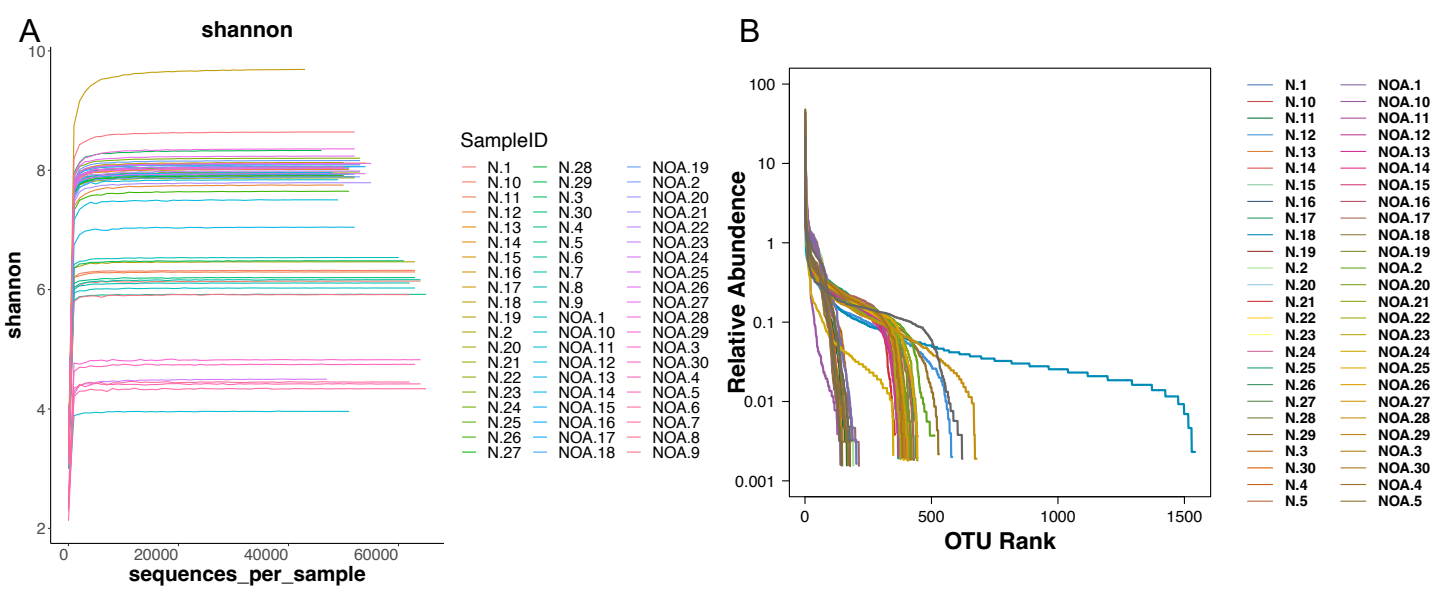

Fig. S1 Dilution curves for individual samples

Supplement: Supplemental file 1 — Fig. S1. Download spectrum.04365-22-s0002.pdf, PDF file, 0.1 MB [file spectrum.04365-22-s0002.pdf]
